# Neuroendocrine Regulation and Neural Circuitry of Parenthood: Integrating Neuropeptides, Brain Receptors, and Maternal Behavior

**DOI:** 10.3390/ijms26189007

**Published:** 2025-09-16

**Authors:** Philippe Leff-Gelman, Gabriela Pellón-Díaz, Ignacio Camacho-Arroyo, Nadia Palomera-Garfias, Mónica Flores-Ramos

**Affiliations:** 1 Instituto Nacional de Perinatología, Ciudad de México 11000, Mexico; pleff2020@gmail.com; 2 Neuroscience Health Foundation, Ciudad de México 04320, Mexico; gabitus@gmail.com; 3 Unidad de Investigación en Reproducción Humana, Instituto Nacional de Perinatología-Facultad de Química, Universidad Nacional Autónoma de México, Ciudad de México 04510, Mexico; camachoarroyo@gmail.com; 4 Escuela Superior de Medicina, Instituto Politécnico Nacional, Ciudad de México11340, Mexico; nadiapalomerag@gmail.com; 5 Instituto Nacional de Psiquiatría Ramón de la Fuente Muñiz, Ciudad de México 14370, Mexico

**Keywords:** maternal behavior, medial preoptic area, peptide hormones, heteroreceptor complexes, cell-signaling systems, mood disorders

## Abstract

Maternal behavior encompasses a range of biologically driven responses whose expression and duration vary across species. Maternal responses rely on robust adaptive changes in the female brain, enabling mothers to engage in caregiving, nourishing, and offspring protection. Morphological and functional changes in the maternal brain enhance sensitivity to offspring cues, eliciting maternal behaviors, rewarding responses, and social processing stimuli essential for parenting. Maternal behavior comprises a range of biological responses that extend beyond basic actions, reflecting a complex, evolutionarily shaped neurobiological adaptation. These behaviors can be broadly categorized into direct behaviors, which are explicitly aimed at the care of the offspring, and indirect behaviors that, overall, ensure the protection, nourishment, and survival of the newborn. The secretion of main neuropeptide hormones, such as oxytocin (OT), prolactin (PRL), and placental lactogens (PLs), during the peripartum period, is relevant for inducing and regulating maternal responses to offspring cues, including suckling behavior. Although PRL is primarily associated with reproductive and parental functions in vertebrates, it also modulates distinct neural functions during pregnancy that extend from lactogenesis to adult neurogenesis, neuroprotection, and neuroplasticity, all of which contribute to preparing the maternal brain for motherhood and parenting interactions. Parvocellular OT-containing neurons in the paraventricular nucleus (PVN) and in the anterior hypothalamic nucleus (AHN) project axon collaterals to the medial preoptic area, which, in turn, projects to the nucleus accumbens (NACC) and lateral habenula (lHb) via the retrorubral field (RRF) and the ventral tegmental area (VTA), which mediate the motivational aspects of maternal responses to offspring cues. The reshaping process of the brain and neural networks implicated in motherhood depends on several factors, such as up- and downregulation of neuronal gene expression of bioactive peptide hormones (i.e., OT, PRL, TIP-39, galanin, spexin, pituitary adenylate cyclase-activating polypeptide (PACAP), corticotropin-releasing hormone (CRH), peptide receptors, and transcription factors (i.e., c-fos and pSTAT)) in target neurons in hypothalamic nuclei, mesolimbic areas, the hippocampus, and the brainstem, which, overall, regulate the expression of maternal behavior to offspring cues, as shown in postpartum female rodents. In this review, we describe the modulatory neuropeptides, the neural networks underlying peptide transmission systems, and cell signaling involved in parenthood. We highlight the dysregulation of neuropeptide hormones and their receptors in the central nervous system in relation to psychiatric disorders.

## 1. Introduction

### 1.1. Motherhood and Parental Behaviors

Motherhood and parental behaviors are expressed in diverse and extensive ways, characterized by nurturing, care, and protection of the offspring [1]. In humans, parenthood requires a long-term commitment and precise planning, shaped by social and cultural contexts that critically influence parent–child interactions [2,3]. Newborns require an extended period of intensive caregiving and protection from male and female parents. In humans, as in certain primate and rodent species, parenthood—defined as the capacity to form a caregiving bond with offspring regardless of biological ties—greatly enhances the nurturing of the young. Given that newborns are nutritionally dependent on lactation, mothers hold a critical caregiving role across all mammalian species, a phenomenon referred to as “maternal behaviors or maternal responses” [4]. Maternal behavior encompasses a range of biological responses that surpass basic actions varying across species. These behaviors can be categorized into direct actions on the offspring (e.g., behaviors related to the young) or indirect interactions that ensure their safety and support lactation needs [4]. Maternal care is not purely instinctual; rather, it requires preparatory changes in the brain driven by placental hormones released into the maternal bloodstream during late pregnancy. This neuroendocrine priming begins before parturition and gradually declines in the postpartum period [4]. Maternal behaviors rely on substantial and adaptive changes in the female brain that enable the expression of caregiving, nursing, and offspring-protective responses [4]. In species with smaller brains, subcortical and limbic neural networks are involved in controlling social and affiliative behaviors that facilitate social communication and reward processing [5,6,7] in response to environmental stimuli. In most mammals, olfaction is their dominant sense, which strongly drives several types of social interactions. In line with this, in animals with smaller brains (rodents, marmosets, and shrews), sensory cues derived from offspring activate maternal responses through the stimulation of the olfactory system and neural pathways projecting to the hypothalamic and mesolimbic regions, which ultimately promote nutrition and survival of the newborn [5,8]. In non-human primates, the olfactory system, along with several limbic structures and their neural circuitry, has been supplanted by neocortical inputs that evolved as a regulatory system implicated in processing sensory cues that elicit adequate biological responses to social and environmental stimuli [1,9,10]. In humans, pregnant women display significant adaptive changes in distinct areas of the brain that allow the female to prepare for motherhood. These changes include a reduction in gray matter volume in specific cortical and subcortical areas, coupled with increased neural connectivity (which enhances neural communication between hypothalamic nuclei and the mesolimbic dopamine pathway) [11,12,13,14]. Moreover, such changes convey neural information demanded to elicit maternal behaviors, rewarding responses, and social processing stimuli needed for parenting in response to offspring’s cues [8,15]. Hence, the ongoing morphological changes occurring in the maternal brain endow females to assume their role as mothers, broadening their sensitivity to their offspring’s cues. The reshaping process of neural networks and brain areas during motherhood is merely dependent on the synthesis and release of several modulatory peptides and trophic factors, and the activation of their specific cell-signaling cascade pathways, which, overall, trigger the maternal care responses required for nourishment, protection, and survival of the offspring [1,2,4]. Therefore, this review outlines the peptide and non-peptide neuronal transmission systems, heteroreceptor complexes, and cell-signaling pathways related to peptide hormones that underscore the expression of maternal behaviors. Lastly, we will address certain dysfunctions in maternal behaviors observed in animal models and their potential connections to human mental health disorders, particularly in relation to prenatal and postpartum affective conditions.

### 1.2. Hormonal Influences on Maternal Behavior

In humans, the peripartum stage is marked by notorious variations in steroid hormone levels, which play a crucial role in the onset and maintenance of maternal behaviors. Throughout pregnancy, serum levels of progesterone (P4) and estradiol (E2) gradually rise. At the time of delivery, the decline in progesterone and the rise in estradiol levels enhance uterine activity, while oxytocin (OT) released from the posterior pituitary promotes uterine muscle contractions [16,17]. These abrupt hormonal oscillations and the changes occurring in the brain neurochemistry significantly impact the functional activity of the maternal brain during late pregnancy and the early postpartum period [16,17]. Following childbirth, prolactin (PRL) levels surge to enhance milk production, while OT stimulates milk ejection in response to the infant’s suckling behavior. These changes in hormone levels are essential, as they synchronize parturition and lactation with the onset of parental behaviors, preparing the mother’s body and brain neural pathways for the adaptive maternal behaviors [2]. Neurons releasing OT from the paraventricular nucleus (PVN) and supraoptic nucleus (SON) are implicated in the onset of maternal behaviors [5,15,18], motivation, bonding [2], and social-related interactions [18,19]. Thus, OT seems to set the “gateway” for maternal behavior and social rewards, relieving anxiety, fostering motivation, encouraging pair bonding, and enabling empathy-promoting comforting behavior [5]. Moreover, during lactation, the secretion of GnRH from hypothalamic neurons is completely inhibited, resulting in a decrease in E2 serum levels, which contributes to the postpartum anestrous phase commonly observed in early lactating female rodents [20]. In the same context, maternal behaviors in female rodents encompass a variety of complex, pup-directed responses, which include retrieving pups, nesting, licking of the pups’ anogenital area, huddling over pups, and adopting nursing postures like kyphosis (a posture adopted by rodents while nursing), in addition to placentophagia, which occurs immediately after delivery [4,5,12]. Other components of the maternal system include PRL and E2, which bind to their cognate receptors and activate their cell-signaling pathways in the medial (mPOA) and lateral preoptic (LPO) areas of the hypothalamus [5]. These regions, together with other neural structures (e.g., cortical areas and thalamic and limbic regions), contribute to the remodeling of the “*maternal brain and its complex neural network system*” [19,21,22]. Furthermore, OT induces a series of behavioral adaptations during the peripartum period, encompassing various elements of maternal care, maternal aggression, improved spatial memory, recognition of offspring (including sensitive auditory processing of offspring vocalizations), reduced anxiety and fear responses, and a diminished neuroendocrine stress reaction (for review, see refs. [2,21]). Interestingly, various studies have demonstrated the endocrine communication between the mother and the conceptus, which appears to be thoroughly mediated by placental lactogens (PLs), and which exerts an important influence on the development of maternal behaviors [23]. Moreover, peculiar studies have detailed interesting observations about the mitogenic activity detected in the cerebrospinal fluid (CSF) of pregnant rats (days 12–21 of gestation), which coincided with a robust increase in placental activity (i.e., metabolic and endocrine functions) and secretion of placental lactogens [23]. This activity was neutralized by administering rodent PL (rPL) type I and II antibodies (Abs), revealing that infusing both rPL-I and rPL-II Abs into the mPOA significantly decreased the latency of maternal responses in nulliparous rats [23]. The maternal neural network enables the swift initiation of maternal behaviors in rodents and mitigates the initial neophobic reactions to pups typically seen in virgin females [24]. Most peptides released from neurons modulate neural pathways through paracrine effects, acting on synaptic, perisynaptic, or extrasynaptic receptors on target cells, thereby impacting their specific cell-signaling systems [2,25]. The precise localization of hormone receptors has been instrumental in dissecting the modulatory effects of brain peptides involved in parenting and motherhood [2,5]. Moreover, maternal adaptive changes in brain structures are contingent on hormone bioactivity, such as PRL secretion (essential for lactation), which is enhanced by inhibiting dopamine (DA) neuronal firing activity and suppressing its release in the dorsomedial arcuate nucleus (dmARC) [2,4,26]. Human studies have provided compelling insights regarding OT’s role in mediating anxiolytic responses. In this context, one notable report indicated that children of depressed mothers with low baseline urine OT levels exhibited a diminished OT response. For instance, blood samples from mothers displaying lower plasma OT levels were associated with a negative OT response in children. Conversely, higher maternal OT levels were associated with unaffected child OT responses, suggesting that maternal OT activity might serve as a buffer against the potential depressive effects experienced by the child [27].

### 1.3. Neural Pathways Involved in Motherhood

**Figure 1:**
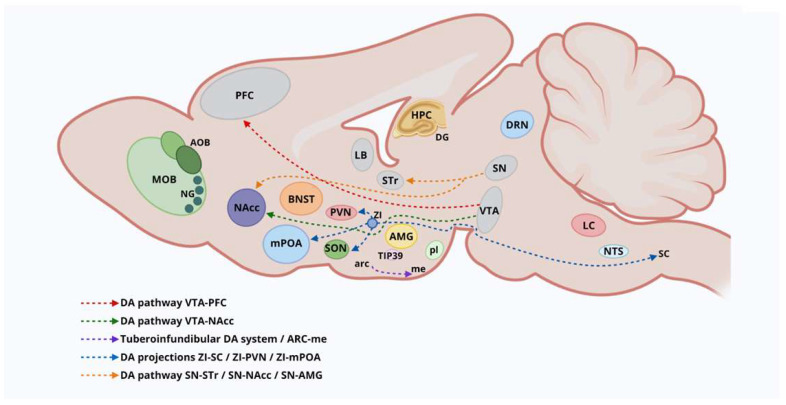
Schematic representation of brain structures and dopamine neural pathways implicated in parenthood and motivational behaviors. The oxytocinergic pathway arises from several hypothalamic nuclei (mPOA, Peri-PVN, and PVN) and regulates maternal behaviors by stimulating the mesolimbic DA pathway (VTA-Nacc-PFC) implicated in motivational care and response. An essential downstream DA neural pathway arises from the ZI and innervates hypothalamic regions, such as the PVN, mPOA, and SON, and extends caudally to the brainstem and SC. In addition, the ARC also projects DA neural information, passing through the tuberoinfundibular area and medial eminence, where the TIP39 peptide appears to localize in this region. Other dopamine routes from the dorsal and ventral striatum may also be implicated in regulating motor activity in parenting behavior. Abbreviations: AOB, accessory olfactory bulb; AMG, amygdala; arc, arcuate (nucleus); BNST, bed nucleus of the stria terminalis; DG, dentate gyrus; DRN, dorsal raphe nucleus; HPC, hippocampus; LC, locus coeruleus; LS, lateral septum; MOB, main olfactory bulb; MOB, main olfactory epithelium; me, medial eminence; mPOA, medial preoptic area; NAcc, nucleus accumbens; NG, necklace glomeruli of olfactory bulb; NTS, nucleus of the solitary tract; OT, oxytocin; PFC, prefrontal cortex; pl, posterior lobe; PVN, paraventricular nucleus; SC, spinal cord; SN, substantia nigra; SON, supraoptic nucleus; STr, striatum; TIP39, tuberoinfundibular peptide 39; VTA, ventral tegmental area (see text for details).

The abrupt onset of maternal behavior observed in mammals such as rodents, primates, and humans concurs with changes in several peptide and non-peptide hormones occurring in late pregnancy and childbirth [12]. However, additional mechanisms have been proposed to explain the elicitation of maternal behavioral responses. For instance, virgin female rats exposed to pups (regardless of the time of exposure) respond with a steady stimulus that evokes significant maternal motivation [28]. Earlier studies identified specific brain regions involved in maternal neuroendocrine activity, such as the PVN and SON, which contain the majority of the brain’s OT neurons [5]. The maternal neural circuitry consists of an output neural projection pathway that extends from the mPOA to the ventral tegmental area (VTA) and the NACC, projecting downstream to hindbrain nuclei [2]. Similarly, several input pathways that directly or indirectly target the mPOA involve mesolimbic regions [i.e., the bed nucleus of the stria terminalis (BNST) and medial amygdala (MeA)] and multisensory systems (i.e., main olfactory and accessory nuclei) [5,7,8] (Figure 1). The olfactory bulb (OB) does not receive direct oxytocinergic projections but is rich in OT receptors, which are activated by neurohumoral OT released into the CSF during childbirth and mating [2]. Sensory information, like olfactory cues (e.g., pheromones), stimulates the main olfactory epithelium (MOE) and intrinsic OB neurons that project a neural pathway to the mPOA, thereby activating maternal behaviors [29,30]. Conversely, cues stimulating the vomeronasal organ (VNO) project downstream to mesolimbic areas, such as the MeA and the BNST, which promote offspring-directed aggressive behaviors in mammalian species [2]. Nevertheless, this aggressive behavior is typically notorious in “sexually” naïve male mice, which diminishes upon cohabitation with a pregnant female. These data suggested changes in the neural chemistry in the VNO [2,30]. Numerous studies have indicated that male mammals possess similar neural structures responsible for driving maternal behavior in females (excluding lactation) and are activated during male parenting, as evidenced in biparental species. These findings purport that cues emerging from pups activate related parenting brain circuitry in male and female brains [2,12,22,30]. In contrast, lesions affecting the olfactory areas (MOB), mPOA, and BNST abolish parental behaviors in both sexes [2,5].

### 1.4. Mesolimbic Dopamine Neural Pathways

The mPOA and VTA are highly responsive to hormones (e.g., E2 and OT), which significantly modulate maternal behaviors (e.g., pup retrieval, nursing, and nest building). Consequently, these hormones require the functional integrity of the downstream-projecting OT neural pathway to the VTA in order to trigger maternal behaviors [12,22,31]. Furthermore, the integrity of the mesolimbic DA pathway is a critical neural component that contributes to the reinforcing properties of pups and the motivational effects associated with motherhood [12,30]. Lesions in the mPOA or temporary inactivation of the VTA eliminate maternal behaviors (e.g., pup retrieval, nursing, and nest building) in postpartum female rodents [22,31]. Taken together, the mesolimbic DA system serves as a downstream target of the mPOA projection neural pathway, which, along with other mesolimbic components of the maternal circuit, is involved in expressing rewarding properties and parental responses, highlighting a preference for pups over other highly reinforcing stimuli (e.g., food and drugs) [12] (Figure 1).

### 1.5. Analyzing Hypothalamic Nuclei and Neural Pathways

Several studies have shown an increased expression of immediate-early genes (i.e., c-fos) [32] and higher levels of brain activity estimated through 2-deoxyglucose [33] in the mPOA of dams exposed to pups [34]. The mPOA has been recognized as a key area regulating social behaviors and homeostatic functions, including reproduction, aggression, and thermoregulation [2]. It is theorized that the mPOA sends neural projections to the NACC—either directly or indirectly via the retrorubral field (RRF) and the VTA—which mediate the motivational aspects of maternal responses to infant cues [2,31]. This circuit appears to be upregulated by projections from the PVN [35], the lateral habenula (lHb) [36], and through serotonergic inputs originating from the dorsal raphe nucleus (DRN) [37] (Figure 1).

Interestingly, the projections of the mPOA to the periaqueductal gray (PAG) and reticular formation via the RRF seem to mediate various motor elements of maternal behaviors [2]. For example, the ventrolateral subdivision of the caudal PAG has been shown to regulate maternal aggression and kyphosis [38,39]. Electrical inhibition and excitotoxic-induced lesions in the mPOA resulted in the curtailment of maternal behaviors, except for those behaviors involved in feeding responses [40]. Conversely, electrical stimulation of the mPOA heightened maternal responsiveness [5,40].

In line with this, in situ hybridization techniques combined with immunolabeling studies for galanin peptide (Gal) and OT within the rat hypothalamus revealed a high density of Gal mRNA neurons in the anterior commissural nucleus (ACN), the mPOA, the medial preoptic nucleus (MPO), and the lateral preoptic area (LPA), including the ventrolateral preoptic area (VLPO), in dams [41]. However, most galanin-immunoreactive neurons (Gal-ir) in the ACN (88%) were found to co-express oxytocin immunoreactivity (OT-ir), and, conversely, most OT-ir cells in the ACN were co-localized with Gal-ir (91%). In this area, Gal-ir neurons were mostly expressed in cell bodies in granule-like structures and in proximal parts of primary dendrites, whereas OT-ir neurons were abundant in secondary dendrites [41]. Interestingly, within the mPOA, a very low percentage of Gal-ir neurons were found to co-express OT (9%) [41].

Interestingly, mPOA-Gal neurons appear to receive several neuronal inputs from at least 20 different neural sites in both male and female mice [42]. mPOA-Gal neurons receive inputs from monoaminergic and neuropeptidergic modulatory areas, the mesolimbic reward system, and pathways associated with pheromone processing, including several hypothalamic areas as well as septal areas involved in emotional states [42]. It is quite interesting to observe that the processing of local mPOA-Gal neurons is modulated by several inputs, such as the monoaminergic- and peptidergic-projecting neural fibers, the dopamine mesolimbic reward system, neural circuits associated with pheromone processing, and neuronal extensions from hypothalamic and septal areas involved in emotional states Moreover, mPOA-Gal neurons do not receive direct neuronal inputs from oxytocin OT magnocellular neurons from the PVN (PVN-OT); instead, AVP-containing neurons within the PVN (PVN-AVP) send abundant axonal collaterals to mPOA-Gal cells, which appear to modulate social behaviors, maternal behavioral responses, and nest building [42].

Moreover, molecular investigations have indicated that approximately 20% of Gal neurons in the mPOA (mPOA-Gal) are involved in integrating the neural pathways that express social and maternal behaviors [2,41,42]. While the mPOA has been considered responsible for controlling paternal behaviors and maternal responses via excitatory (glutamatergic) projecting neurons [2], mPOA-Gal axon fibers are primarily GABAergic [2,41]. These findings suggest that the mPOA exerts a distinctive form of parental control through inhibition or disinhibition of lower brain nuclei [2,18,21].

### 1.6. Oxytocin Neurons: Magnocellular Neurosecretory Cells

The magnocellular neurosecretory cells are mainly located within the SON and PVN in the anterior hypothalamus. These regions represent key areas responsible for secreting both OT and vasopressin (AVP) into the bloodstream [43,44]. Additionally, they encompass parvocellular neurons that project to the median eminence (ME) and other brain regions [41,43]. Magnocellular neurosecretory cells synthesize various neuropeptides far beyond OT and AVP, which include apelin, Gal, the neuroendocrine regulatory peptides, the pituitary adenylate cyclase-activating polypeptide (PACAP), and secretin [44,45,46,47,48], which are stored and released from large dense-core vesicles (LDCVs) [44].

### 1.7. Oxytocinergic Neural Pathways

**Figure 2:**
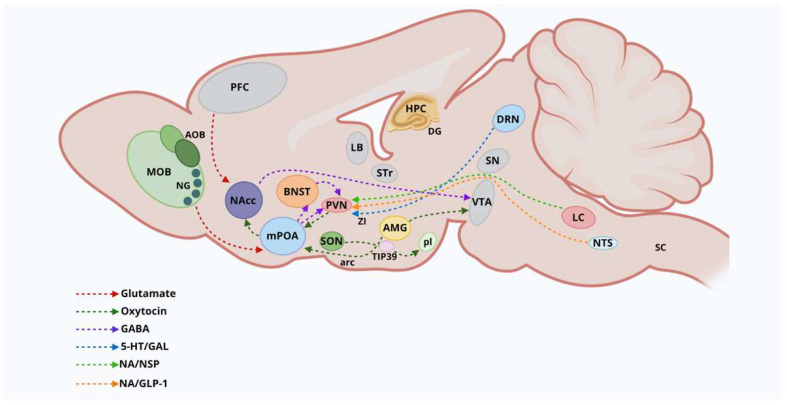
Schematic representation of brain structures and neural pathways implicated in parenthood and motivational behaviors. As shown, glutamatergic pathways emerging from cortical areas, the hippocampus, and the main olfactory bulb stimulate limbic structures involved in maternal behaviors. Projection fibers emerging from the DRN, LC, and NTS appear to participate in the regulation of the motor components and motivation responses to pup cues. Monoamines (DA, NA, and 5-HT) and peptides (OT, GAL, NSP, and GLP-1) are involved in the complex neural wiring that drives and modulates several aspects of parenting and maternal behaviors in response to offspring stimuli (see text for details). The locations of several brain regions are not exact for illustration purposes. Abbreviations: AOB, accessory olfactory bulb; AMG, amygdala; arc, arcuate (nucleus); BNST, bed nucleus of the stria terminalis; DG, dentate gyrus; DRN, dorsal raphe nucleus; HPC, hippocampus; LC, locus coeruleus; LS, lateral septum; MOB, main olfactory bulb; MOB, main olfactory epithelium; me, medial eminence; mPOA, medial preoptic area; NAcc, nucleus accumbens; NG, necklace glomeruli of olfactory bulb; NSP, neuroendocrine-specific protein; NTS, nucleus of the solitary tract; OT, oxytocin; PFC, prefrontal cortex; pl, posterior lobe; PVN, paraventricular nucleus; SC, spinal cord; SN, substantia nigra; SON, supraoptic nucleus; STr, striatum; TIP39, tuberoinfundibular peptide 39; VTA, ventral tegmental area (see text for details).

Anatomical mapping using OT-Cre mice and viruses targeting OT cells has revealed that PVN-OT circuitry neural pathways project to nine functional circuits that control cognition, brain state, and somatic visceral response [49,50]. As such, PVN-OT neurons project axon collaterals to functionally relevant brain structures, such as the thalamus, cortex, amygdala, striatum, and hippocampus [49,50]. Most OT axon fibers extending from extrahypothalamic regions—such as the amygdala (AMG), BNST, lateral septum (LS), and septal nuclei—target hypothalamic OT nuclei, along with axon fibers emerging from the brainstem, thalamic structures, and cortical areas, have been implicated in eliciting parental behaviors across species [49]. In this context, both BNST and the mPOA represent critical maternal brain core regions that promote the expression of maternal behaviors through OT secretion from neuronal cells [18,19,50,51,52] (Figure 2).

Approximately 80% of brain regions surrounding the ventricular system in mammals, including the subarachnoid space, express OT receptors (OTRs), which are vital for the manifestation of social and maternal behavioral responses [53,54]. The release of OT amplifies synchronized neural activity during suckling and lactation [49,54,55]. Additionally, parvocellular OT-containing neurons in the PVN and anterior hypothalamic nucleus (AHN) project extensive axon fibers to the brainstem and spinal cord [49].

### 1.8. Oxytocin Receptors

From the early postpartum period, OTRs undergo a dramatic upregulation and increased receptor expression in the mPOA, which promotes the maternal behavioral responses [19,52]. Animal studies showed a species-specific variability in OTR distribution across the brain, which not only affects parental and social behaviors [49,53] but also promotes lasting effects on brain organization and behavior [52,54]. Sexual dimorphism is evident in OTR expression, with higher levels found in the olfactory bulb, piriform cortex, CA2 hippocampal area, and left auditory cortex of the female mouse brain [53]. Moreover, several studies indicate that AVP receptors (AVPR1A) also mediate parental responses, in addition to OT receptors [49,56]. Both OT and AVP receptors exhibit a distinct and well-organized spatial distribution within the brain scenery, and their segregation bears significant physiological outcomes for peptide receptor interactions [49,55,56]. Based on the distribution of receptors in the mammalian brain, activation of either the OTR-Gαq/11-PLCβ signaling pathway (Gαq/11→PLCβ→PIP2→IP3 + DAG→PKC) or the OTR-Gαq/11-MAPK→ERK1/2 downstream pathway in target neurons promotes a variety of biological activities, including modulation of social behavior, aggression, parturition, synaptic development, circuit plasticity (spatial memory) [57,58], and anxiolytic effects through interactions with opioid receptors (e.g., μOR and κOR) [59].

### 1.9. Oxytocin and Dopamine Heterodimeric Receptor Complexes

Dopaminergic neurons significantly enhance OT release in limbic areas, as DA receptors are expressed on OT neurons [60,61]. Conversely, OT has been shown to elevate DA levels in the mPOA, amygdala, and VTA, where OTRs are expressed [60]. Recent research has demonstrated heterodimerization between D2R and OTR in striatal neurons, alongside the expression of D1R and D3R receptors on OT neurons [60,61]. These molecular formations appear to regulate OT-mediated responses in social interactions and emotional behaviors [61,62]. Previous studies have indicated that the coactivation of D2R and OTRs is critical for social attachment. These studies revealed that striatal OTRs promote pair-bonding in female prairie voles, while D2Rs mediate social attachment interactions [63]. Such behavioral responses are facilitated by the formation and activity of D2R-OTR heteroreceptor complexes in both the dorsal striatum and the NACC core [62].

Allosteric mechanisms mediated through receptor–receptor interactions, as shown by OTR heterocomplexes, significantly foster D2R recognition (increasing the high-affinity state and density of D2Rs) and D2R Gi/o coupling mechanisms [62]. These findings shed light on the dynamics of D2R-OTR heterocomplexes in DA neurons. For example, OT produces an inhibition of the AC-PKA-pCREB signaling cascade mediated by the D2R-like agonist quinpirole, while quinpirole significantly increases the signaling of the PLCβ-IP3-calcineurin pathway [64]. These results provide valuable insights into the role of OT and DA interactions in social bonding and their anxiolytic effects within the amygdala of rats [62,64].

### 1.10. Oxytocin Neuromodulation

Animal studies showed that OT neuromodulation regulates synaptic inhibitory activity in target cells. OT and its associated cell-signaling pathway promote cellular disinhibition at postsynaptic sites across various brain regions [49,53]. This modulation leads to the activation of local inhibitory circuits, such as GABAergic interneurons, enhancing signal strength and neuronal activity related to parental and social information processing [49]. Notably, OT fibers innervating dopaminergic structures in the midbrain influence DA neuron-firing rates, boosting the excitability of the VTA-DA neural pathway associated with maternal behavioral responses [49,53,65]. Furthermore, oxytocinergic fibers originating from the SON and PVN nuclei of the hypothalamus extend axon collaterals to multiple brain regions, driving parental and maternal behaviors in mammals (such as rodents) [49,52].

### 1.11. Brain Peptides Influencing Maternal Behavior

Over recent decades, numerous neuropeptides and their receptors have been identified, enhancing our understanding of their involvement in mood-related behaviors influenced by stress in animal research. Some of these modulatory peptides [i.e., hypocretins and melanin-concentrating hormone (MCH)] [66,67] and related neural circuitry involved in lactation and parenting behaviors [68] have been reported to modulate both parenting and maternal behaviors, which are not described in detail in the present review.

### 1.12. Urocortins

Urocortin peptides (UCN1, UCN2, and UCN3) exhibit varying binding properties to the corticotropin-releasing hormone receptor 1 (CRHR1) and corticotropin-releasing hormone receptor 2 (CRHR2) receptors, suggesting that CRH is not the sole ligand for these receptors, apart from the non-mammalian sauvagine [69]. For example, UCN1 displays a high binding affinity for both CRHR receptors, exhibiting a functional role in stress responses [70]. Mice exposed to chronic stress paradigms showed an increase in c-Fos expression in UCN1-producing neurons, and maternal separation of rat pups produced a prolonged insensitivity of UCN1 neurons in response to acute stress in adult offspring [71]. Moreover, UCN1 mRNA levels were found to be increased in brain samples from suicide victims compared with non-depressed individuals [72]. In addition, behavioral studies focused on the UCN2 peptide demonstrated that intracerebroventricular (ICV) administration of UCN2 in mice and rats produced high levels of anxiety responses, which correlated with an increase in c-Fos expression in DRN-5-HT neurons projecting to the PVN [69]. Furthermore, UCN3, also known as stresscopin, selectively activates the CRHR2 receptor subtype. While UCN3’s central distribution indicates a primary role in energy homeostasis, animal studies have shown that ICV administration of UCN3 can lower anxiety levels in the light–dark test in mice, whereas restraint stress elevates UCN3 expression in neuronal cells within hypothalamic and limbic regions. It has been suggested that UCN3 and its peptide fragments have anti-depressive effects in mice, with a CRHR2 antagonist inhibiting this mood-enhancing response [69].

### 1.13. Endogenous Opioid Peptides

It is commonly understood that the three types of opioid receptors (μ, δ, and κ) are activated by endogenous peptides derived from three distinct precursors: proopiomelanocortin (POMC), proenkephalin (ProENK), and prodynorphin (ProDYN) [73]. The post-translational processing of these precursors results in over 20 peptides with opioid receptor activity [i.e., β-endorphin (1-31) (β-end 31), Met-Enk, and dynorphin A (1-17) (Dyn A17)], which bind to their selective cognate receptors, namely, μOR, δOR, and κOR, respectively. These opioid receptors are expressed in several target cells in the CNS and peripheral tissues [73]. However, recent studies showed that opioid peptides may be less selective than previously thought, effectively binding and signaling through all three opioid receptors [73].

### 1.14. Opioid Receptors Modulating OT Neural Pathways

Both exogenous opiates and endogenous opioids have been shown to interact with the OT neural system [74]. Previous studies showed that μOR and κOR are expressed in OT neurons [75], suggesting that these interactions occur on hypothalamic magnocellular neurons. These receptors were shown to exert presynaptic inhibition on input-synapsing OT neurons, as well as in axons extending to the pituitary [75]. Although these reports assumed that endogenous opioids block the electrical and secretory activities of OT neurons, further studies revealed that such responses depended on the animal’s physiological state [76]. For instance, basal OT release, either into the circulatory system or locally within the hypothalamus, was significantly inhibited by opioid peptides in magnocellular neurons of pregnant rats but not in virgin females [77]. Additionally, animal studies revealed that swim stress triggered a substantial neuronal release of OT in both the PVN and SON regions of the hypothalamus in pregnant rats, which was inhibited by naloxone. In contrast, in virgin rats, endogenous opioids steadily stimulated OT release from PVN neurons [57,77].

Further studies showed that the potent opioid-mediated inhibition of OT neurons in pregnant dams was mediated by μOR located in somatodendritic sites in OT neurons. Such inhibition was observed during a short time period prior to the onset of parturition. This inhibitory effect facilitates the synthesis and accumulation of OT in secretory granules, enabling its release during delivery and promoting maternal behavioral responses [57].

Although these studies, performed in experimental animals to explore the regulation of OT neurons via opioid receptors’ bioactivity, are still ongoing, there are still several unanswered questions that require further exploration.

### 1.15. Galanin and Spexin Neural Peptides

Galanin (GAL) is a regulatory neuropeptide with a wide distribution in vertebrates and the mammalian brain [78,79]. GAL is co-expressed with various classical neurotransmitters, including acetylcholine (Ach), serotonin (5-HT), glutamate (GLU), GABA, noradrenaline (NA), and DA [79]. The GAL family currently includes Gal, galanin-like peptide (GALP), galanin-message associated peptide (GMAP), and alarin. These structurally related neuropeptides are associated with a broad range of biological and pathological functions [78,79]. The GAL peptide exerts its central and peripheral actions through three G-protein-coupled receptors, galr1-3 [79]. Notably, Gal demonstrates a higher affinity for galr1 and galr2 receptors [78], whereas GALP has a preference for binding to the galr3 receptor [78,79]. Like GAL, GALP is abundantly present in the mammalian brain and neuroendocrine tissues [78,79]. Additionally, a novel endogenous 14-amino-acid regulatory peptide, named spexin (SPX), has been isolated from the rodent CNS [80,81,82]. This peptide shows high binding affinities for both galr2 and galr3 receptors, with the greatest affinity for galr3 [82]. SPX has been recognized as an endocrine-signaling peptide exerting numerous functions, such as regulation of energy balance, weight loss, fatty acid uptake, glucose homeostasis, nociception, reproduction, and feeding behavior [83], including social behaviors [82,83]. SPX has been detected in various mouse tissues, including the brain, HPA axis, gastrointestinal tract, liver, gonads, and adrenal glands [83,84]. Furthermore, in vivo and in vitro studies have demonstrated that Gal and its receptors (e.g., galr1 and galr2) modulate the immune system by enhancing the secretion of pro-inflammatory cytokines and chemokines from human macrophages [85].

Previous research indicated that OT neurons in the PVN and SON co-express Gal [2,41,42]. Notably, 91% of OT-immunoreactive cells in the ACN were found in neurons that also expressed Gal-immunoreactivity (Gal-ir), while approximately 9% of OT-ir neurons co-expressed Gal-ir in the mPOA. These findings posited that both peptide hormones may be co-expressed in distinct neuronal populations, despite their subcellular localizations [42]. In addition, cellular and behavioral studies using c-fos immunolabeling showed that ablation of neurons expressing GAL-ir in the mPOA induced a significant reduction in maternal behavior and preferences for pups, promoting aggressive behaviors toward the offspring [83]. Moreover, optogenetic activation of preoptic Gal neurons led to increased responses in pup care behaviors and grooming behavior [2,42]. In agreement, mPOA-Gal neurons co-expressing OT-ir in the rat brain, including Gal-ir neurons in the ACN, have been linked to maternal behaviors [2,42]. It is important to note that the ACN-magnocellular neurons expressing OT-ir are considered a subdivision of the PVN and part of the OT neural system [42] (Figure 2). Moreover, lesions targeting mPOA-OT neurons, while sparing other hypothalamic nuclei, were associated with diminished parental behaviors in mother rats [36,42,49], indicating that mPOA-OT neurons are crucial for driving and regulating maternal behavioral responses [22,42,49].

Previous studies showed that stimulation of neural inputs during suckling enhances the activity of OT- and GAL-containing neurons in the mPOA, neurons that are critical for maternal responsiveness [22]. Nipple stimulation triggers the release of prolactin (PRL) from the pituitary, initiating lactation in mothers [23] and reducing maternal anxious behaviors during the postpartum period [23]

Interestingly, GAL neurons in the ACN expressed the STAT5 transcription factor (pSTAT5) [42]. This factor was shown to mediate cellular responses of PRL in target neurons, contributing to the PRL-induced anxiolytic effects [42]. Most GAL neurons in the mPOA showed no expression or activation of pSTAT5, indicating that PRL does not influence mPOA cells [42]. As such, the PRL neural pathway transmitting suckling information relies on the posterior intralaminar complex of the thalamus (PIL) and the subthalamic nuclei [5,42]. This area contains the tuberoinfundibular peptide 39 (TIP39), a neuropeptide involved in mediating maternal motivation during suckling [5,42]. Cellular studies demonstrated that TIP39-containing neurons from the PIL project their axon terminals to mPOA-GAL-containing neurons [5,42]. Furthermore, TIP39 axon terminals contain and release glutamate (GLU). Such information suggested that PIL-TIP39 neurons establish excitatory connections with mPOA-Gal neurons, involved in processing sensory information during lactation [5]. Neurons containing TIP39 in the PIL were found to modulate the activity of mPOA-Gal neurons during maternal care and motivation [5,42]. In a similar context, related studies revealed that a subgroup of TIP39 neurons sends axon terminals to the arcuate nucleus, suggesting that thalamic TIP39 neurons convey suckling information from the mPOA to the ARC. These results posited that TIP39 neurons promote PRL release in female rats during pup suckling [42,86] (Figure 2).

### 1.16. Galanin, Serotonin, and Zinc Heterodimeric Receptor Complexes

Previous studies showed that in membrane preparations from the ventral limbic cortex (VLCx), Gal peptide (1–29) in the nanomolar range reduced the binding affinity of [3H]-5HT1A agonist binding sites at postjunctional sites, suggesting that Gal decreased 5-HT1A recognition for its cognate receptor in this brain region [63,87,88]. This antagonistic mechanism of the GalR1-5HT1A heteroreceptor complex suggests that this complex could promote pro-depressive effects [89]. Experimental findings in genetically modified mice showed an increased expression and density of galr1 and galr2 in serotonergic neurons in the DRN [63,87]. Interestingly, galr1 homodimerization and dimer internalization were detected in transfected cultured cells, as well as with the galr1-5-HT1A heteroreceptor complex observed in HEK 293 cells [63,87,90]. These studies revealed an antagonistic allosteric receptor–receptor interaction and the coactivation of the galr1 and 5-HT1A protomers, each one coupled to the Gi/o-MAPK signaling pathway, showing an increased stimulation of MAPK activity in HEK293 cells [91]. However, these studies suggested that in depression, the antagonistic allosteric mechanism on the galr1-5HT1A heteroreceptor complex becomes dysfunctional, producing disturbances in the mesolimbic serotoninergic neurotransmission [63,87,91]. Moreover, cellular studies demonstrated that zinc (Zn^2+^) binding to its cognate gpr39 receptor produced a disruption of the galr1-5HT1A heteroreceptor conformation. In this context, gpr39 forms a dynamic heterotrimeric receptor complex (galr1-5HT1A-gpr39), which displays a diversity of signaling transduction pathways in target cells [92]. This heterotrimeric receptor complex appears to represent an intermediate and unstable conformational state that depends on Zn^2+^ [92]. These studies showed that gpr39’s interaction with the 5HT1A-galr1 heteroreceptor promoted a change in the molecular equilibrium toward the gpr39-5HT1A heteroreceptor conformation, thus supporting the molecular mechanisms of the antidepressant response induced by zinc [87,93]. In line with these findings, additional studies have demonstrated that under high zinc concentrations, the 5HT1A receptor does not interact with galr1, whereas the 5HT1A-gpr39 heteroreceptor complex resulted in the principal molecular conformation in cells [92].

In line with this, different studies revealed that the N-terminal Gal fragment (1–15) displays high-affinity binding sites in the rat hippocampus (dhipp), prefrontal cortex (PFCx), and striatum (Str) [63,87], suggesting that N-terminal Gal fragment-binding sites promote conformational changes in galr1 and galr2 isoreceptors, enabling the formation of galr1–galr2 heteroreceptor complexes [94]. Moreover, related studies showed that Gal (1–15) was able to induce effects in 5-HT1A receptors in the raphe–hippocampal-projecting serotonergic neural system, facilitating the conformation of the galr1-5-HT1A and galr2-5-HT1A heteroreceptor complexes, respectively, in the dorsal hippocampus (dhipp) and the DRN [63,87,94]. Similar studies described the formation of heterotrimeric complexes between galr1, galr2, and 5-HT1A heteroreceptors in postjunctional sites in target cells or at prejunctional synaptic terminals [95]. In addition, several pieces of evidence demonstrated that Gal (1–15) may promote a disbalance of the signaling pathway of the galr1–galr2 heterodimer, enhancing the activation of the Gi/o signaling system through the galr1 protomer, whereas the Gq/11 signaling of the galr2 protomer produced no significant effects. This bias in the signaling systems of the galr1–galr2 isoreceptors showed that application of galr1-synthetic agonists led to the expression of depression-like bioactivity [94].

### 1.17. Tuberoinfundibular Peptide 39

**Figure 3:**
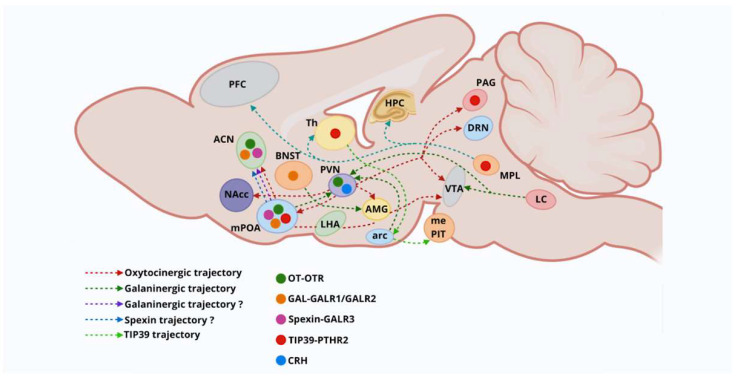
Representation of the peptidergic neural pathways involved in parenting and maternal behaviors. The figure depicts the neural pathways and inputs for several peptides distributed along the rostrocaudal regions and nuclei of the rodent brain. Color symbols (⬤) represent the peptides binding to their cognate receptor or receptors. Lines depict putative projection neural pathways, some peptides, and their cognate receptors in target cells that could be involved in parenting and maternal behaviors. Most brain areas depicted herewith have been described in Figure 1, except for the other structures and peptide transmitters not described (see text for details). Locations of brain areas are not precisely exact for illustration purposes. Abbreviations: HIP, hippocampus; LHA, lateral hypothalamic area; MPL, medial paralemniscal nucleus; PAG, periaqueductal gray; PIL, posterior intralaminar area of the thalamus; PIT, pituitary; Th, thalamus; TIP39, tuberoinfundibular peptide 39.

TIP39, or parathyroid hormone-2 (PTH2), represents the endogenous ligand of the orphan parathyroid hormone 2-receptor (PTH2R) [5,96]. The cellular expression of TIP39 is detected in two specific brain sites: the periventricular gray (PVG)–subparafascicular nucleus (SPF) in the medial thalamus and the medial paralemniscal nucleus (MPL) in the lateral pons [5,42], in addition to the PIL, considered the third brain area of TIP39 expression. This peptide was not detected in the adult brain [5,42]. Thus, TIP39 is no longer present in the PIL in the early postpartum period and is undetectable in the adult male brain [5,96,97]. Nonetheless, TIP39 mRNA levels are very low in the adult female rat [5,97]. In contrast, a huge increase in TIP39 and its encoding mRNA was found in the PIL in mothers from the early postpartum period, lasting until the weaning of pups [5,42,97]. Thus, TIP39 levels remain elevated during the lactation period [5,42,97]. During maternity, TIP39 expression within the MPL in the pons is induced to a lesser extent than in the PIL [5,97] (Figure 3).

### 1.18. Prolactin Modulatory Bioactivities

Prolactin (PRL) is an ancient regulatory polypeptide hormone [4,98] shown to display pleiotropic functions that go far beyond its roles in reproduction and lactogenesis [99]. Although PRL is mainly produced by lactotroph cells in the pituitary, different reports showed that extra-pituitary tissues also produce and release PRL, such as the immune system, endothelial cells, the reproductive system, and the CNS [99]. Thus, PRL modulates the immune system, growth, metabolic functions, osmoregulation, and several brain functions [100,101]. In spite of this, PRL bioactivity is merely related to reproduction and parental behaviors, which have been conserved along evolution (i.e., fish, birds, and mammals) [102]. PRL modulates neural functions such as adult neurogenesis, neuroprotection, and neuroplasticity during pregnancy, thus facilitating the preparation of the brain for parenthood [100,103].

Moreover, PRL permeates the brain through a saturable carrier-mediated transport system [4], and the PRL concentration in the CSF was found to parallel PRL levels in the blood [4]. Interestingly, in addition to the placental-derived lactogens (i.e., hPLs) acting in the brain, several reports documented that PRL is synthesized and released into the CSF from the epithelial cells lining the choroid plexus [4,103].

During pregnancy, the human placental lactogen (hPL) (a member of the gene family of the growth hormone (GH), which shares 85% homology with the pituitary GH) is produced by the placental syncytiotrophoblasts [99]. hPL is detected in the maternal circulation in humans after 5–7 weeks of gestation, increasing its concentrations throughout pregnancy, reaching its peak concentrations (5.0–7.0 µg/mL) at term. hPL displays PRL-like properties, which are responsible for its lactogenic and mammogenic responses, although its role in human lactation is still unclear [98]. hPL-mediating PRL-like bioactivities are merely due to the hormone’s ability to bind to both PRL and GH receptors, suggesting that both GHR and PRLR may form a dynamic heterodimeric complex [100]. Both PRL and hPL are relevant to functions related to mammogenesis, lactogenesis, galactopoiesis, and galactokinesis, which are essential to enhance lactation in the offspring. As extensively described, PRL is ascribed as the predominant galactopoietic hormone, while oxytocin (OT) represents the main galactokinetic mediator in mammalian species [104]. Furthermore, different studies showed that OT, serotonin (5-HT), opioids, histamine, substance P (SP), and arginine–leucine (Arg-Leu) regulate PRL secretion from the adenohypophysis through autocrine and paracrine-related mechanisms [5].

### 1.19. Prolactin Cell-Signaling System

Prolactin receptor (PRLR) has been shown to be expressed in a wide range of tissues throughout the body, including the brain [4]. PRLR has been detected in the subventricular zone (SVZ) of the hippocampus in mice, in addition to the median eminence, the subfornical organ, and the choroid plexus (CP), hypothalamus, epithalamus, amygdala, and brainstem [4,105,106]. PRL receptor is structurally related to the class 1 cytokine receptor superfamily, which is encoded in humans by the *PRLR* gene on chromosome 5p13-14 [4,107]. This receptor consists of a conserved PRL-binding extracellular domain, a 24-amino-acid-long transmembrane domain, and a cytoplasmic domain coupled to signaling transduction proteins, and shown to vary among cells and species [4,107]. Thus, PRLR comprises several receptor isoforms, which vary in size and function [4]. PRL binding to its extracellular domain promotes the dimerization of PRLR and activation of the Janus kinase 2 (JAK2) signaling system, which activates the phosphorylation and translocation of signal transducer and activator of transcription 5 (pSTAT5) to the nucleus, affecting gene transcription [4,107,108]. Two homologous transcription factors of pSTAT5 were identified in tissues, such as the STAT5a and the STAT5b isoforms [4,109]. For instance, in the mammary gland, PRLR signaling is mediated via STAT5a [4,110,111], whereas in the hypothalamus, PRL is mediated via activation of STAT5b [4,110]. Immunolabeling studies showed that both pSTAT5 isoforms mediate PRL bioactivities in distinct brain areas, particularly those involved in expression of maternal behaviors and lactation [4]. Nonetheless, similar studies showed that PRL mediates its functions by signaling other transduction pathways, either through the mitogen-activated protein kinase (MAPK) and the signal-regulated kinase (ERK) systems, which appear to regulate antiapoptotic and proliferative responses in neuroendocrine cells (i.e., β-cells in the pancreas) [98,111].

### 1.20. Prolactin Regulates Neural Functions

Several reports have shown that PRL directly influences neuronal activity and cell-related functions. As previously reported, an important mechanism that regulates PRL secretion is the short-loop feedback that regulates the hormone release. As shown, PRL stimulates the activity of tuberoinfundibular dopamine (TIDA) neurons in the ARC of the hypothalamus, enhancing an increased release of DA, which leads to the subsequent inhibition of PRL secretion from the pituitary [4]. In this context, previous studies demonstrated a high number (>90% in rats; ~80% in mice) of TIDA neurons that express the PRLR [4,112]. PRLR stimulation promotes a switch in the phasic firing pattern of DA neurons to a tonic firing response [113,114]. PRL induces a fast increase in the cell-firing activity in a large number (~70%) of TIDA neurons [115], showing that PRL stimulates a high-voltage component that signals the phosphoinositide 3-kinase (PI3K) pathway, which requires the activation of BK K+ channels [116]. In addition, a low-voltage component consisting of a mixed cationic transient receptor potential (TRP) channel was detected upon stimulation of the PRL receptor [116,117]. Interestingly, in females, PRL has also been found to sensitize TRP channels in sensory neurons, implicating them in the regulation of pain responses [117,118]. Contrary to the PRL-inducing effects in TIDA neurons, PRL stimulation activity in the brain was observed in a subset of neurons that express the PRL receptor (~30%) in the PVN and the mPOA, and the mechanism by which PRL induces its neuronal effects is merely through gene transcription [114].

### 1.21. Prolactin Induces Maternal Behaviors

Animal studies have shown that PRL induces crucial acute effects on neurons in the mPOA [116]. Compared with the ARC nucleus, PRL-induced effects were observed in a much smaller subpopulation (~25%) of mPOA cells [116], suggesting that PRL regulates the cells primarily through transcriptional signaling [112,119]. The PVN of the hypothalamus was also shown to drive maternal behaviors and organize Prlr expression in target cells [119]. In line with this, previous studies showed that with in situ administration of PRL into the PVN, a small group of OT neurons were inhibited in both virgin and pregnant rats, but not in lactating rodents [120,121,122]. Conversely, upon examining PVN cells expressing the Prlr in the mouse brain, PRL-inducing effects were mixed in content, enhancing either inhibitory or excitatory responses in males, virgin females, and lactating females [116]. However, as with the mPOA, these direct PRL-mediated effects on neuronal activity were detected in a subpopulation of Prlr-expressing PVN neurons (~30%) [116], providing evidence that gene transcription appears to be the primary mechanism of PRL-mediated responses in the PVN [116,120].

As mentioned earlier, offspring-directed behaviors are crucially relevant to enhancing maternal responses (i.e., retrieval of offspring or pups, licking, grooming, and nursing/crouching behaviors) and the survival of the newborn. These behavioral responses are mediated via PRL secretion from pituitary lactotrophs [4,104]. In line with this, behavioral studies using a genetic model of Prlr-knockout mice revealed that in the absence of Prlr signaling, both retrieval and crouching responses over pups were sharply disrupted in virgin female mice and heterozygote dams [121]. However, ICV administration of PRL elicited a dose-dependent pup retrieval behavior in nulliparous female rats [122], as well as direct infusions of PRL or placental lactogen into the mPOA [122,123]. Such experiments revealed that the mPOA represents the key neural site for PRL-induced maternal behaviors [4,122,123].

Finally, and crucially, with regard to maternal nest building, nesting behavior is an energetically dependent bodily mechanism that ensures offspring survival, as shown in altricial species, due to the fact that newborn species exhibit a reduced capacity in regulating temperature and locomotion. This maternal behavior has been shown to be preserved among vertebrate and mammalian species. For instance, pregnant female mice build a brood nest during early gestation, which is triggered by pup cues that stimulate the maternal olfactory bulb [124]. Thus, maternal nesting appears to be a PRL-mediated response via Prlr signaling on mPOA neurons [125]. Nonetheless, lactogenic hormones may also contribute to the expression of this behavior [126].

## 2. Stress Responses

### 2.1. CRH and HPA Axis Activity

The corticotropin-releasing hormone system and the HPA axis have been defined as the “final common pathway” and represent the stress response system to multiple stressors [127]. This stress response system consists of neurosecretory neurons localized in the medial parvocellular region of the PVN that secrete both corticotropin-releasing hormone (CRH) and arginine vasopressin (AVP) in the median eminence (ME) in response to stress [128]. These peptide hormones induce the secretion of ACTH from “pituitary corticotropes” via the CRH-binding receptor—CRHR1—and the CRHR-signaling pathway (via the cAMP-PKA-CREB cascade), including the AVP1B receptor and the activation of the PKC-signaling system, which supports the CRH bioactivity in the ACTH release from adenohypophysis. CRH promotes transcription of the POMC gene, which encodes both ACTH and β-lipotropin [128]. The secretion of ACTH enhances the synthesis and release of glucocorticoids (i.e., corticosteroids) from the adrenal cortex and increases levels of mRNA-encoding enzymes responsible for the steroidogenic pathway. As a result, end products (CORT) mediate negative feedback (limiting glucocorticoid release) on the HPA axis, reducing PVN neural activity and ACTH release, and thereby enhancing a temporal constraint in stress-inducing hormonal secretion [129].

However, several studies have provided evidence that AVP acts as a stronger stimulus on ACTH secretion from pituitary corticotropes [130] and that splanchnic nerves mediate the sympathetic potentiation of ACTH-induced CORT release following acute and chronic stress [129,131,132]. In addition, pro-inflammatory cytokines also enhance the release of adrenal CORT, thus supporting that alternative pathways and mechanisms may operate in CORT secretion from adrenals besides ACTH [133]. Although the HPA axis plays a central role in regulating several behavioral processes and stress responses [134], an overt increase in HPA axis reactivity may hamper maternal behaviors and lactation [124], among several other brain functions [134].

### 2.2. Stress Effects on Maternal Behavior

Pregnancy and lactation drastically reduce HPA axis function in mammalian species, including humans [4,135]. Stressors during pregnancy and lactation have been reported to enhance poor pregnancy outcomes, increasing the risk for PPD, in addition to the mother renouncing the offspring [4,136,137]. Stressors during pregnancy may trigger negative effects on fetal neurodevelopment and growth, leading to important changes in brain programming, which ultimately may lead to gender-dependent heightened stress responses in the offspring, associated with an altered epigenetic profile [138,139,140]. Therefore, a reduced bioactivity of the HPA axis to impinging stressors is crucially important to allow the neuroadaptive changes to motherhood to assure the welfare of both mother and offspring [4,141]. Animal studies have shown that suppression of the neuroendocrine-stress response system during lactation in female mice (induced through a restraint or endotoxin stressor) led to a significant reduction in both ACTH and CORT secretion when compared with virgin females [142]. Additionally, both temperature and exercise-inducing stress were found to be dampened during pregnancy and lactation in humans [4,136].

Moreover, recent studies have shown that acute stress induced an increase in CRHR1 levels in the rostral anteroventral periventricular nucleus (AVPV/PeN) (a brain region involved in neuroendocrine stress responses and maternal behaviors), including the PVN, the mPOA, and the BNST in postpartum female mice compared with nulliparous mice [143]. Interestingly, similar studies have shown that AVPV/PeN-CRHR1 neurons and PVN neurons co-express the glucocorticoid receptor (GR), whose activity could be linked to the increased crhr1 levels detected in these brain regions in animals exposed to stress [143]. Such an increase in crhr1 levels was found to be associated with an increase in anxious behavioral responses in postpartum rats [144]. Interestingly, female mice exhibiting a deletion of crhr1 showed deficits in nursing behavior [145], whereas pharmacological activation of crhr1 in the BNST and mPOA showed a similar significant reduction in lactating responses in the postpartum mice [146].

### 2.3. Prolactin-Regulating Stress Responses

HPA axis hyperactivity and hypercortisolemia have been demonstrated in animal models of stress-inducing altered behaviors and in pregnant women exhibiting perinatal depression [147]. Animal studies demonstrated that CRH mRNA levels in the PVN were significantly lower during pregnancy and lactation in female mice compared with virgin controls [4,148]. Conversely, recent findings showed that pup removal (which decreases the suckling-induced PRL secretion) enhanced an increase in PVN-CRH mRNA levels in female mice, which were re-established to low lactating levels upon PRL administration [148], supporting previous studies showing that PRL regulates CRH secretion from hypothalamic explants [4,149]. These studies are in line with other findings that showed that PRL displays dose-dependent anxiolytic effects in non-pregnant rats [4,150]. In addition, PRL administration induced a reduction in ACTH and corticosterone secretion in virgin rats exposed to anxiety-like stressors [151]. Such PRL-induced anxiolytic effects could be due to reduced c-fos immunoreactivity in the PVN in animals exposed to chronic stress [151]. Nonetheless, these effects do not appear to depend exclusively on a PRL direct mechanism, as PRLR mRNA is not expressed by PVN-CRH neurons [152]. Furthermore, PRL did not show a direct activation of PVN-CRH neurons based on pSTAT5 immunoreactivity in virgin and lactating mice and rats, respectively [152,153]. However, experiments based on gene manipulation in lactating rats so as to induce a downregulation of the expression of the PRL receptor in neuronal cells showed that the decrease in prlr expression led to an important increase in stress-inducing anxious behavior, associated with an increase in ACTH levels compared with control female rats [152,153]. Overall, these studies showed evidence of the fundamental role of PRL-regulating maternal adaptive behaviors, albeit that the exact brain area where this hormone exerts its anxiolytic effects is still unknown [4,152,153]. Nonetheless, both PRL and the prlr-signaling system highlight the importance of how this system regulates HPA axis activity in stress-related disorders [140,152,153], not to mention the relevance of this peptide hormone, whose treatment could ameliorate or prevent the risk of developing anxiety in vulnerable pregnant women, which ultimately impacts mother–child interactions [154,155].

### 2.4. Oxytocin-Modulating Stress Responses

Besides PRL-enhancing anxiolytic effects in animals and humans [4], OT has been shown to elicit maternal behavioral responses in extrahypothalamic brain regions, where OT projection neural pathways have been shown to mediate such responses in various species [57,156]. During late pregnancy and the early postpartum period, the behavioral adaptations to motherhood and lactation were associated with an increase in activity of OT neural components in the mammalian brain [57,157]. These adaptive changes include the increased synthesis of OT in MNCs to meet the demands of OT secretion and OT vascular concentrations required during parturition, birth, and suckling stimuli [158,159]. In addition, an increase in OTR expression and OTR density was detected in neural circuits in the PVN, BNST, medial (MS) and lateral septum (LS), dorsal hippocampus (dhipp), mPOA, VTA, basolateral (BLA), central (CeA) amygdala, MeA, PFCx, and anterior olfactory nucleus (AOB), brain areas reported to be crucial in promoting and modulating maternal responses to offspring [57,160,161]. Such broad axonal projections to the forebrain were found in mammalian species (including reptiles), where OTR-signaling pathways were shown to be highly activated in the peripartum period [19,162,163,164] and in lactation [158,165]. Thus, OT-induced neuroadaptive changes in neural function in the peripartum period enhance the behavioral responses to the newborn, which include maternal care and protection, enhanced spatial memory, offspring recognition, and the development of sensitive auditory processing in offspring vocalization. These maternal behaviors reduce anxiety and fear responses and decrease the HPA axis activity to impinging stressors [57]. In this context, both physiological and behavioral aspects triggered by OTR-signaling systems are essential to establish the adaptive maternal responses needed to enhance an adequate attachment that will enhance the growth of the undeveloped offspring [161].

Notably, the anxiolytic and anti-stress responses promoted by OT are substantially traced in the peripartum, which facilitates the optimal conditions to prepare pregnant females for motherhood [166]. Pharmacological studies shed interesting insights into the neural mechanisms by which OTR mediates the maternal responses in rodents. For instance, ICV administration of OT in steroid-primed virgin female rats produced a short delay in maternal responses [167], whereas the ICV administration of an OTR antagonist or in situ infusion of a synthetic antagonist into the mPOA, or lesioning the PVN, provoked a delayed onset of maternal behaviors (i.e., pup retrieval and nursing posture over pups) in postpartum damns [34,57,163,168]. This delay in the expression of maternal behaviors did not impede the cell release of OT, showing that OT levels measured in the extracellular space remained constant [19,168]. Furthermore, these OT-dependent responses were associated with a robust increase in OTR expression in hypothalamic and mesolimbic regions, as demonstrated by the OTR-increased activity in the mPOA in lactating female rats [163].

An interesting aspect to highlight is that OTR-binding activity within the dHipp appears to be crucially relevant for spatial memory linked to maternal behavior [57,169]. As such, in lactating mice, OT infusion enhanced a long-term spatial learning process, most likely mediated by the OTR-signaling MAPK-pCREB cascade pathway, as extensively demonstrated to promote a robust LTP response in the hippocampal formation [169]. This crucial finding highlights that female rats exhibiting improved spatial memory and hippocampal-related learning and memory processing during motherhood could explain how dams return to the nest location when searching for food at more distant spots [57,159].

Some studies demonstrated another “scheme” by which OT modulates maternal responses in mice. In this study, OT neurons were transfected through a virus encoding a specific variant of the rhodopsin channel under the control of the *Oxt gene*. The functional expression of the *Oxt* gene revealed that optogenetic stimulation of OT neurons within the PVN significantly decreased the latency in virgin female mice to express maternal behaviors [57]. Similarly, related studies revealed that optogenetic stimulation of a small dopaminergic–neuronal cluster located in the anteroventral periventricular nucleus (AVPV) of the hypothalamus led to an increase in OT secretion and higher maternal responsiveness. These neurons were found to relay monosynaptic inputs to OT neurons to the PVN and the mPOA, resulting in the upregulation of maternal responses [57]. More interesting are the studies reported by Marlin et al. [170], which showed that the preferential lateralization in OTR expression in the left auditory cortex (A1 and AII) is crucially important for the onset of pup retrieval behavior once the offspring starts emitting ultrasonic vocalization [171]. These authors described that the left auditory cortex comprises an extension of the OT-sensitive circuitry required for the onset, but not the maintenance, of maternal behavior responses [170]; in addition, OT increases the neuronal sensitivity, processing, and conversion of “feeble” responses in virgin female rodents into a sturdy maternal response in the postpartum period [170].

In the same context, experiments in genetically manipulated female rats, displaying high levels of neuronal OTR expression in the PVN, mPOA, LS, CeA, and BNST, led to an increase in pup-licking/grooming behaviors, showing that female rats were more susceptible to respond to pup cues [1]. These studies showed that DA and OT interactions in the VTA, NACC, and mPOA were responsible for the induction of maternal motivation and for the prolonged maternal attraction to pups throughout the postpartum period [161]. Interestingly, behavioral studies revealed that in both OT- and OTR-knockout mice, the “maternal arched back nursing posture” was significantly enhanced in response to pup-suckling [171]. However, mutant mice displayed an impairment in OT- and OTR-signaling systems, which impeded pup retrieval and pup-licking behaviors in female mice, displaying an increased latency in both the onset and maintenance of maternal motivation responses [171,172].

It is worth noting that OTR expression in the PFCx was observed to be relevant in the expression of maternal responses and anxiety-related behaviors [57,173]. In line with this, in situ administration of OT into the medial prefrontal cortex (mPFCx) produced a significant decrease in anxious behavioral responses in both male and female rats [174]. Conversely, administration of a selective OTR antagonist led to an impairment of maternal care, enhancing aggressive behaviors toward pups [174].

### 2.5. Galanin and Spexin Peptides: Pro- and Anti-Stress Responses

The galanin (1–29) peptide and structurally related Gal peptides (e.g., GAMP, GALP, and alarin) have been extensively reported to regulate numerous physiological processes, like feeding, pain, anterior pituitary hormone regulation, energy and osmotic homeostasis, reproduction, and cognition, in addition to pathophysiological mechanisms through interactions with distinct G protein-coupled receptors, namely, gal1r, gal2r, and gal3r isoreceptors, which signal multiple transduction pathways on target cells (see above) [69,78,79]. This peptide was found to be widely distributed in the rat central nervous system and peripheral tissues in vertebrate species [79] and shown to regulate mainly stress-inducing disorders [69,175]. In line with this, different studies reported that the bilateral administration of Gal into the CeA induced an anxiolytic effect, which was blocked through the Gal antagonist M40 in rats [175,176]. In a similar context, Gal-mediated an anxiolytic effect was demonstrated in transgenic mice overexpressing the Gal peptide, and ICV administration of yohimbine (an antagonist of α1, α2, D2R, and 5-HT1B/ID/2A/2B receptors) showed an impaired induction of anxiogenic responses [175,176]. These effects were due to the reduced levels of NA and GLU in both the amygdala and HPc [176]. In a similar context, administration of selective galr3 antagonists (e.g., SNAP 37889 and SNAP 398299) evoked anxiolytic behavioral responses in rats, mice, and guinea pigs, owing to reduced Gal-stimulatory activity on this receptor [177]. Moreover, stimulation of gal1r and gal3r elicited anxiogenic and depressive responses, whereas activation of galr2 induced opposite effects [178]. Confirmation of such behaviors was demonstrated with mutant gal2r KO mice, who displayed reduced anxiety-like behaviors [178,179] or through genetically modified mice overexpressing gal2r, who exhibited a decrease in anxiety- and depression-like behaviors in chronic restraint stress paradigms. However, mice displaying high levels of Gal and gal1r expression showed opposite responses [180,181]. Such behavioral responses were reversed through antidepressant treatment (SSRIs). Such treatment induced a significant increase in gal2r expression levels in the hypothalamus and limbic regions [178,181].

Studies in human subjects also showed the role of Gal and their signaling pathways in the pathogenesis of affective disorders [78,79]. In line with this, genetic studies showed that the rs1042577 SNP within the 3′ untranslated region of the Gal gene and the haplotype analysis of the rs948854 C_rs4432027_C allele were found to be associated with high levels of anxiety in a large population of inpatients displaying MDD [182].

Spexin (SPX) is the most newly discovered neuropeptide among the family of Gal bioactive peptides found in the mammalian brain. These Gal-related peptides have been shown to play a key role in the regulation of metabolism, endocrine (pancreatic β-cell) function, energy homeostasis, mood, and behavior, as shown in zebrafish, rodents, and humans [182].

Furthermore, Gal-signaling systems and the SPX-binding galr2 isoreceptor were found to decrease or suppress insulin secretion in animal models (except in humans), showing that SPX exhibited a potent anorectic, analgesic, anxiolytic, and antidepressant-like effect in rodents [183]. These SPX-mediated effects were owed to the modulatory activity of SPX in serotonergic neurons in the DRN involved in mood control, and in POMC-producing neurons in hypothalamic areas involved in appetite and body weight control [183]. Thus, it appears that SPX-binding galr2 promotes anxiolytic responses, whereas Gal, depending on its binding receptors, may elicit either antidepressant or pro-depressive behavioral responses [183].

## 3. Psychiatric Disorders

### 3.1. Affective Disorders in Postpartum Period

Both bodily and mental well-being in mothers entering the postpartum period represent a crucial factor to enhance the proper conditions required for the offspring’s development and normal growth [184]. In the same line, recent reports showed that the prevalence of postpartum depression (PPD) oscillates from 13% to 19%, with a higher risk of developing major depression during the first six months after parturition [185]. Risk factors are mostly the same as those reported in major depressive disorder (MDD), with the exception of postpartum-specific factors, such as sensitivity to hormonal changes [186]. Recent studies have reported that the prevalence (0–24 weeks) of postpartum anxiety symptoms is 13.7% and 8.4% for anxiety disorders [184], showing the negative effects of anxiety on mother–infant interactions, feeding practices, infant temperament, and social–emotional development [187].

Moreover, life stressors (i.e., history of depression, low maternal self-efficacy, poor current health of the mother, and no breastfeeding after PP-3 weeks) have a greater impact on the risk of developing PPD, and these factors may arise before women get pregnant, during pregnancy, or afterward [185]. However, stressors leading to postpartum anxiety appear to be quite different from those reported for PPD (i.e., history of depression, preterm birth, negative experience during delivery, uncontrolled infant crying, low maternal self-efficacy, low partner support, and poor current maternal health during the first week of postpartum) [184].

With regard to medical support, psychological interventions showed effectiveness in both individual and group sessions. Despite pharmacological treatment for PPD revealing an improvement in depressive symptoms, enhancing maternal care and breastfeeding, controlled trials revealed no differences in outcomes between patients under antidepressant medication and those treated with a placebo [185].

### 3.2. Oxytocin in Mood Disorders

**Figure 4:**
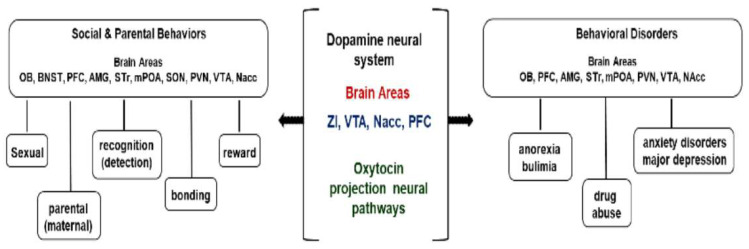
Interaction of dopaminergic, oxytocinergic, and neural systems involved in social, parental behaviors, and psychiatric disorders. Dopamine- and oxytocin-interacting systems drive several socio-affiliative components and parental behaviors, including the galanin transmission system. Dysfunction of these neural systems, which includes neurotransmitters, receptors, and cellular-signaling systems, may lead to subsequent mood-related disorders and behaviors. Brain oxytocinergic neurons are activated through the incerto-hypothalamic (ZI) dopamine neural input and mesolimbic dopamine pathways. The neuronal release of OT from hypothalamic and limbic regions, and Gal- or SPX-binding galr2 or galr3 receptors (anxiolytic and non-depressant) in limbic structures, integrates the complex neural circuitry that drives social and parental behaviors. Disruptions or dysfunctions of these neurochemical pathways could partially underlie some of the pathophysiologic mechanisms that contribute to organic functions in addition to the negative effects and adverse responses that impinge on the array or molding of social parameters, parenting behaviors, maternal care, and motivation to offspring cues. These altered behaviors in males and females could lead to the development of psychiatric disorders and altered behaviors (see text for more details). Abbreviations: NAcc, nucleus accumbens; ZI, zona incerta; mPOA, medial preoptic nucleus; PVN, paraventricular nucleus; SON, supraoptic nucleus; AMG, amygdala; VTA, ventral tegmental area; HC, hippocampus; OB, olfactory bulb; STr, striatum; PFC, prefrontal cortex; BNST, bed nucleus of stria terminalis.

Subjects diagnosed with MDD meet criteria for comorbid anxiety disorder [57,186] and are usually associated with severe social dysfunctions, which include social withdrawal, social fear, impaired social recognition, and aggression. As extensively reported, OT exerts a crucial role in the pathology process of MDD. In humans, both anxiolytic and prosocial effects of OT mediation could be used as a potential therapeutic benefit in patients exhibiting severe affective disorders [187] (Figure 4).

Electroconvulsive therapy (ECT) represents one of the most effective treatments for drug-resistant MDD, merely due to its fast response. ECT was shown to promote an increase in plasma OT levels [57,188], albeit a clear relationship between ECT, plasma OT, and clinical outcome has not been fully reported [189]. This could be due to the stressful events that ECT elicits despite the response caused by the acute rise in plasma OT levels.

Similarly, clinical studies reported that MDD patients displayed positive correlations between plasma OT and the severity of symptom clusters found in major depression. In line with this, significant correlations were found between OT levels in the blood and the severity of depressive symptoms in individuals exposed to a clinician-rated instrument, such as the affiliation-associated imagery [190] used to explore impulsiveness and negative emotionality [57,190] and/or between reward dependency and novelty seeking, as assessed in a temperament and character inventory [191] (Figure 4).

However, generally speaking, plasma OT in most MDD patients assessed under basal conditions was found to be inconclusive, showing that OT does not represent a reliable biomarker of MDD, despite the number of studies that measured OT concentrations in the CSF of MDD patients [190,191]. Nonetheless, quantification of OT in the CSF appears to be a potentially reliable lab tool to assess the link between different psychiatric phenotypes [191]. Moreover, postmortem studies evaluating the number of hypothalamic OT neurons and OT mRNA levels showed a consistent increase in the activity of the OT system in depressive subjects, particularly in PVN-OT-ir neurons, which were found to be increased in tissue samples of both MDD and bipolar subjects compared with controls [57,192].

Moreover, in situ hybridization studies revealed an increase in OT mRNA levels in the PVN in melancholic depressive patients compared with non-melancholic individuals [193]. These studies showed a trend toward increased OT mRNA levels in the PVN of the MDD group compared with age-matched controls, as detected by quantitative PCR in snap-frozen brain tissue using a laser capture microscopy [57,193].

With regard to OT treatment in the context of postpartum depression, similar effects were observed when compared with SSRIs [57]. A single study showed that women exposed to an IV administration with a synthetic OT surrogate during delivery (used to accelerate labor and birth) were tested 12 months postpartum for major depression. Interestingly, these women, with or without a history of depressive disorders, exhibited a higher relative risk of developing MDD or a general anxiety disorder (GAD) after being treated with OT during delivery compared with women who did not receive the OT surrogate [194]. Hence, OT treatment during delivery needs to be considered carefully owing to the potential risks of developing psychiatric disorders in vulnerable women and its effects on the newborn or child, rendering OT treatment a suboptimal choice in vulnerable women with a depression background [57,194].

In contrast, a double-blind, randomized, within-subject control study on intranasal OT administration focusing on caregiving behaviors, mood, and physiology in mothers with (subclinical) PPD showed a significant increase in maternal positive regard for the child and self-reported positive affect, showing no effects on maternal sensitivity, negative mood, or physiological responses. This study described that OT’s therapeutic use should be considered in PPD severity or childhood trauma [195].

Altogether, evidence from animal and human studies led to the proposal of the key role of the OT system in the pathophysiology of MDD, despite its potential therapeutic benefit shown at least in some populations of MDD subjects and on some symptoms related to MDD [57]. Nonetheless, the role of the OT system in areas related to behavioral and neuroendocrine regulation and its anxiolytic and anti-stress-inducing effects, including social behaviors and social reward, render the brain OT system a promising candidate to explore possible novel pharmacological therapeutics, as the OT system interacts with various classical neurotransmitter (e.g., 5-HT, DA, and NA) and neuropeptide systems (CRH and opioid peptides) relevant to major depression [57] (Figure 4).

### 3.3. Neural Peptide Systems in Psychiatric Disorders

In the past decades, several neuropeptide families have been discovered and have been shown to display neuromodulatory bioactivities in synapses containing classic small-molecule neurotransmitters. These neuropeptides have highlighted the interest in psychiatry and psychoneuroendocrinology, foretelling the potential clinical relevance in the treatment of stress-inducing mood disorders [69]. As described above, several neuropeptides and their binding receptors in target cells were found to be directly or indirectly affected by stress, as depicted above in animal models of stress [69].

Studies of postmortem brain samples of suicide victims reported that both neuropeptides and/or their cognate receptors could be suitable targets in the development of novel neuropharmacons. Thus, in the last few years, clinical studies have started to assess the effects of potential candidate drugs targeting neuropeptide receptors, thus influencing stress-inducing behavioral responses [69]. A few examples are described below.

### 3.4. NPY and NPY Receptors in Mood-Related Disorders

NPY and its cognate receptor subtypes (Y1–Y5 receptors) have been extensively studied in stress-inducing maladaptive behaviors [69,196]. This peptide belongs to a family comprising neuropeptide Y (NPY), pancreatic polypeptides, and peptide YY. NPY displays a wide distribution in the CNS, exerting various physiological functions, particularly in stress-inducing altered behaviors [69].

Animal studies using a post-traumatic stress disorder (PTSD) animal-related paradigm showed that the administration of a selective NPY-Y1-receptor antagonist produced an increase in predator stress responses, which was reversed after treatment with the peptide antagonist [197].

In line with this, mice overexpressing NPY-Y1 receptors in the hippocampus showed a reduced anxiety phenotype, supporting that the Y1 receptor subtype mediates anxiolytic responses. In contrast, behavioral studies revealed that the in situ injection of an NPY-Y2 receptor agonist into the amygdala elicited increased anxiety [198]. In addition, knockout mice displaying a deletion of the NPY-Y2 receptor displayed a significant reduction in anxiety- and depression-like behaviors, opposite to that shown with the NPY-Y1 receptor [198].

In line with this, NPY-Y5 receptor antagonists (e.g., Lu AA33810) displayed antidepressant and anxiolytic effects in rat models of stress sensitivity [199]. These results provide evidence that NPY-Y1-Y2 receptor subtypes mediate anxiolytic or anxiogenic responses in a brain region-dependent manner via activation of their cell-signaling cascade pathways [69].

### 3.5. PACAP-PAC1 Receptor in Psychiatric Disorders

Several authors have posited that both the pituitary adenylate–cyclase-activating polypeptide (PACAP) and its cognate PAC1 receptor (expressed in neurons within the BNST) are not crucially relevant for anxious behaviors. However, in knockout mice expressing a deletion of the PAC1 receptor, mutant mice displayed reduced anxiety-like behavioral responses [69,200] as well as increased locomotor activity and an increase in immobility time in the forced swim test (FST), suggesting that the neuronal expression of the PAC1 receptor in neuronal cells upregulates anxious behaviors [200].

Interestingly, PACAP-deficient mice showed no connection to the induced increase in CRH mRNA levels in PVN neurons [201]. Nonetheless, these animals showed a sharp increase in c-Fos expression in several brain areas (e.g., BSNT, EW, VLS, and DRN) [202], in addition to an abrupt glucocorticoid response [201].

In a similar context, human studies revealed that the PACAP/PAC1 receptor upregulated the production of the protein DISC1 (shown to be disrupted in schizophrenia) [203], in addition to demonstrating significant associations between SNPs in the PACAP gene and depression [204] and the PAC1 receptor polymorphism in PTSD in female patients [205,206].

### 3.6. Gal and Gal Receptors in Mood Disorders

As depicted above, Gal induces a depression-like phenotype and is shown to be reversed by Gal receptor antagonism [69,176,179]. Pharmacological studies revealed that antagonists binding to gal1r and gal3r blocked depression-like behaviors, whereas gal2r inhibitors displayed opposing effects [207]. Moreover, gal2r-overexpressing mice exhibited reduced depressive behavior [179]. Mice exposed to a restraint stress paradigm induced an increase in anxiety and depression-like behavioral responses, associated with an elevation in both Gal peptide and gal1r expression in the hippocampal formation [69,180].

SSRI treatment showed a counteraction of behavioral responses, inducing a reduction in both Gal and gal2r expression in the brain [69], in addition to the increase in Gal-binding sites in the DRN and a decrease in Gal fiber density in the hippocampus and hypothalamus. Interestingly, these changes were associated with alterations in both CRH and NPY systems in limbic and hypothalamic regions [69].

It is interesting to note that intranasal administration of a SPX-based galr2 synthetic agonist elicited a potent anxiolytic effect, besides an appetite-suppressive response in rats [82]. As mentioned earlier, most studies have purported galr2 as an antidepressant, whereas Gal-binding galr1 or galr3 promoted pro-depressive effects associated with major changes in the serotonergic system [87,91]. Moreover, as galr3 showed a higher binding affinity for SPX than Gal [80,81,82], it may be assumed that the galr3-signaling system and bioactivity may result from SPX [82,87].

Finally, studies in humans have provided clear evidence of the relevance of Gal and its cognate receptors in the pathogenesis of mood disorders [78,79,83]. A genome-wide screening study showed sharp associations in SNPs (rs2156464) between the GAL gene and major depression [208] and SNPs (rs948854) associated with both anxiety disorder, MDD, and HPA axis overactivity in female patients [209].

## 4. Conclusions

Parental behavior is an essential biological phenomenon that guarantees the survival of offspring species, as shown in different forms throughout the animal kingdom. Parents’ attention influences the child’s neurodevelopment, particularly in the infant’s cognitive function. Neglect or child abuse has been shown to predispose children to the development of psychiatric disorders in adulthood. Both parenting and social behaviors in mammals are modulated by cortical, mesolimbic, and hypothalamic brain structures and their operating neural circuits that are finely tuned and regulated to promote parental care and motivation.

As a result of parturition, PRL levels rapidly increase to support milk production, and OT stimulates milk ejection in response to the infant’s suckling. These hormonal changes are crucial because they coordinate parturition and lactation with the onset of motherhood, preparing, thus, the mother’s body, neural networks, and brain cells for maternal care [2]. The dendrites of the magnocellular neurons of the SON and PVN are the source of very large amounts of OT and AVP released within the brain.

OT appears to “gate” the social reward-related behaviors, reducing anxiety and facilitating motivation and bonding, empathy-based consolation behavior, and, in mammalian species with small brains, olfactory-related memories. Moreover, the maternal neural circuitry consists of an “outlet” projection pathway extending from the mPOA to the VTA and NAcc, projecting axon fibers downstream to the hindbrain as well. This mPOA projection neural pathway is an essential “neural component” for the expression of rewarding properties and maternal responses, implying the preference for pups versus other highly reinforcing stimuli (i.e., food and drugs).

Moreover, both PRL, E2, and their cell-signaling pathways, which are highly expressed in the mPOA and LPO, including cortical areas and thalamic and limbic regions, shape and build up the “maternal brain and its neural network system”. These brain regions, driven by several peptide hormones [i.e., PRL, OT, placental lactogens (PLs), TIP39, and Gal], prepare the maternal brain to express pup-directed behaviors, such as pup retrieval, nesting, anogenital licking of pups, huddling and crouching over pups, and nursing postures like kyphosis that appear immediately after parturition.

Interestingly, these peptide hormones bind their specific cognate receptors via the conformation of heteroreceptor complexes, as shown for OTR and D2R, 5-HT1A-GalR1, and 5-HT1A-GPR39 heterodimeric complexes, whose biased cell-signaling pathway may promote and favor the behavioral adaptations during the peripartum period, and which include several aspects of maternal care, maternal aggression, improved spatial memory, offspring recognition, including sensitive auditory processing of offspring vocalization, reduced anxiety and fear responses, and an attenuated neuroendocrine stress response.

Thus, motherhood in mammals requires the reshaping of the brain (brain plasticity) and activation of a complex neural network system driven by mPOA-OT and mPOA-GAL neurons within the hypothalamus. This network and operating neurons may endow the maternal brain as a “brain-commanding structure”, regulating parental behaviors, maternal care, and motivational responses.

In humans, pregnant mothers display significant plastic changes in several brain regions that enhance the preparation of females to motherhood. Brain changes include a decrease in gray matter volume in cortical and subcortical regions (i.e., the hypothalamus) associated with increased neural connectivity (i.e., from the hypothalamus nuclei to the mesolimbic DA neural pathway, which leads to adaptive maternal behaviors, rewarding responses, and cortical-conveying social processing of signals or cues engaged in parenting.

With regard to psychopathology, clinical studies showed associations between clusters of symptoms found in MDD and circulating OT levels, in addition to increased OT mRNA levels in PVN-OT neurons in postmortem brain samples of MD, BD, and melancholic depressive subjects. However, several peptide systems described herein have been shown in stress-inducing mood disorders and are associated with specific SNPs in genes encoding the OT propeptide protein precursor. Thus, treatment of psychiatric disorders in the perinatal period is crucial to enhance the appropriate maternal behaviors and pair-bonding responses, facilitating both parenting and mother–infant interactions.

Overall, motherhood and parenting is a complex repertoire of paternal and maternal behaviors that requires adaptive modifications of the brain and activation of widely spread neural networks, associated with the release of numerous modulatory neuropeptides and neurohormones from somatodendritic synapses that modulate mainly OT and PRL bioactivities (via activation of their cell-signaling cascade pathways and gene transcription) in central and peripheral target cells, during parturition and the postpartum period, to promote the maternal care, protection, and survival of the newborn.

## 5. Perspectives

Maternal mental well-being in the postpartum period is a determinant condition for the mother’s health and for the normal development of the newborn. Based on the reports describing the high prevalence of PPD (with high risk of developing PPD during the first six months after parturition), in addition to postpartum anxiety symptoms and anxiety disorders, it becomes quite clear that PPD and PP anxiety require medical attention, psychological support, and antidepressant treatment (e.g., SSRIs, NSRIs, and esketamine).

The development of agonists or antagonists targeting specific receptors (i.e., galr1, galr2, OTR, and npy-y1r) in the CNS may be a great opportunity to achieve a fast recovery from PPD and maternal anxiety in order to counteract the negative PPD-inducing effects on pair bonding and attachment, which may lead to psychiatric disorders during child development. Thus, a key combination of medical attention, psychotherapeutic intervention, and pharmacological treatment may represent the best choice to achieve both the mother’s and child’s well-being and a healthy attachment, preventing or ameliorating the mother’s affective disorder and associated symptoms that may impact the child’s neurodevelopment, with future negative and severe outcomes.
